# A Report on the Progress of Vegetable Physiology during the Year 1837

**Published:** 1840-07

**Authors:** 


Art. IX.—*A Report on the Progress of Vegetable Physiology during the year* 1837. By F. J. F. Meyen, m.d., Professor of Botany in the University of Berlin. Translated from the German by William Francis, a.l.s.—*London,* 1839. 8vo, pp. 160.

The appearance of this translation has been delayed by a cause which we are sorry to learn—the want of a sufficient number of subscribers to cover the expense of printing it; and the same cause, if it continue to operate, will prevent the translator from pursuing his useful labours by giving the subsequent Reports of the same learned botanist to the English public. We have on so many occasions directed the attention of our readers to the importance of this department of science that we need not now enlarge upon it; and we shall only state that we know of no means of keeping pace with the rapid progress of vegetable physiology, and especially with those continental researches of which but a small part would otherwise find their way to this country, at all to be compared in facility with the study of the admirable Annual Reports of Professor Meyen. And to those who are not extremely conversant with the German language, we strongly recommend the employment of such a faithful and skilfully-executed translation as that of Mr. Francis, as more likely to give them correct and definite notions of the author's meaning than the perusal of the original.

